# Diet of Infants and Young Children

**Published:** 1882-08

**Authors:** 


### BOOK NOTICES, REVIEWS, ETC.

Diet of Infants and Young Children. By J. C. Morgan, M. D. pp. 50. Price, 25 cents. Philadelphia, 1882.

In these fifty pages, Dr. Morgan gives a brief account of some of the many "foods" kindly prepared for suffering babes by *philanthropic* persons. The book is written for mothers, and will doubtless be of service to them.

